# SF-6D Normative Values Among Patients Undergoing Bariatric Surgery: Results Based on Real-World Evidence from the Scandinavian Obesity Surgery Registry (SOReg)

**DOI:** 10.1007/s11695-023-07024-0

**Published:** 2024-01-08

**Authors:** Sun Sun, Erik Stenberg, Nan Luo, Karl A. Franklin, Lars Lindholm, Klas-Göran Salén, Yang Cao

**Affiliations:** 1 Department of Epidemiology and Global Health, Umeå University, 901 87 Umeå, Sweden; 2 Department of Surgery, Faculty of Medicine and Health, Örebro University, 701 85 Örebro, Sweden; 3 Saw Swee Hock School of Public Health, National University of Singapore, Singapore, Singapore; 4 Department of Surgical and Perioperative Sciences, Surgery, Umeå University, Umeå, Sweden; 5 Clinical Epidemiology and Biostatistics, School of Medical Sciences, Faculty of Medicine and Health, Örebro University, 701 82 Örebro, Sweden; 6 Unit of Integrative Epidemiology, Institute of Environmental Medicine, Karolinska Institutet, 171 77 Stockholm, Sweden

**Keywords:** Bariatric surgery, SF-6D, Normative value, Quality-adjusted life years, Health preference, Real-world data

## Abstract

**Background:**

The SF-6D index can be used to calculate quality-adjusted life years in economic evaluations, which is required by reimbursement agencies and national advisory bodies, including the Swedish ones. However, despite that SF-36 has been largely applied among patients undergoing bariatric surgery, almost no study has accessed the *short form six-dimensions (SF-6D)* after bariatric surgery.

**Aim:**

To establish normative values for the SF-6D index among patients undergoing bariatric surgery.

**Materials and Methods:**

All patients who received bariatric surgery in Sweden between 2011–01-01 and 2019–03-31 were obtained from the Scandinavian Obesity Surgery Registry (SOReg). Information includes patients’ sociodemographic characteristics, details regarding the procedure, and postsurgical conditions. The SF-36 is applied at baseline and at follow-up years 1, 2, and 5. The multiple sequential imputation method was applied to handle missingness on SF-6D items. Based on the UK tariff, the SF-6D preference scores were calculated. The normative values for the mean (SD) SF-6D index were reported by timepoint and surgical complications for men and women, respectively. Multivariate analyses were applied to investigate how the SF-6D index is associated with timepoint, controlling for age, sex, BMI, and comorbidities in a stepwise manner.

**Results:**

The SF-6D index increased at 1 year relative to baseline and was roughly maintained at the same level at 2 years. The normative value of the SF-6D index can be used in economic evaluations for bariatric surgery.

**Graphical Abstract:**

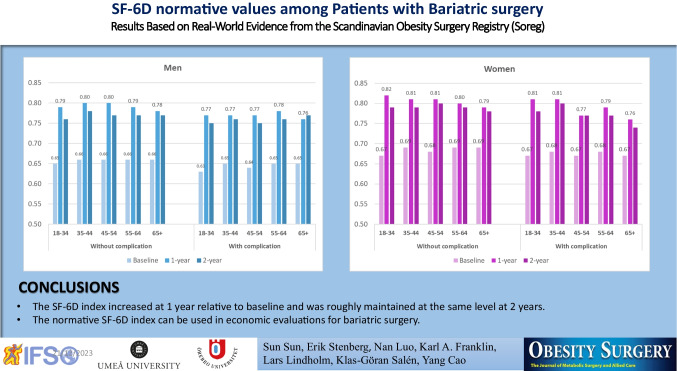

**Supplementary Information:**

The online version contains supplementary material available at 10.1007/s11695-023-07024-0.

## Background

The prevalence of obesity has increased over time, especially in North America and Europe, with 30% of women and 24% of men affected by obesity in the USA [1]. Obesity is associated with significant mortality and reduced health-related quality of life (HRQoL). People with obesity run an increased risk of developing and worsening diabetes mellitus, cardiovascular disease, and cancer. Obesity also imposes a large economic burden on the individual and on families and the society [2] with an estimated impact of 2.8% of the global gross domestic product (GDP) [2]. Management of obesity involves lifestyle modification [3, 4], pharmacotherapy [3, 5], and bariatric surgery [3, 5]. Bariatric surgery is the most effective treatment for weight reduction, and it has also been shown to improve obesity-related complications including diabetes mellitus [6], cardiovascular disease [7], and life expectancy [8].

The most important endpoint for individuals undergoing treatment is the improvement in Health-related quality of life (HRQoL). HRQoL is an important measure as it provides a comprehensive understanding of the patient’s health status and the impact of healthcare interventions on their quality of life. It is used to monitor the effectiveness of healthcare interventions and to evaluate the cost-effectiveness of healthcare programs [9]. Quality-adjusted life years (QALYs) is a measure of disease burden that combines the quality and quantity of life, which is calculated by multiplying the duration of time spent in a health state by the HRQoL weight (i.e., utility score) associated with a certain health state [10]. The Short Form-36 (SF-36) is the most commonly used HRQoL instrument [11–13]; however, HRQoL weight cannot be derived directly from SF-36 scores. The Short Form 6 Dimension (SF-6D) algorithm enables the calculation of HRQoL weights based on SF-36 data, which allows the calculation of QALYS and cost-effectiveness analysis of health interventions [14]. It is used by health technology assessment agencies around the world, including in Australia, Canada, China, Ireland, Japan, Netherlands, Norway, Spain, and the USA [15].

Many studies have reported results from SF-36 in patients following bariatric surgery; however, only a few reported results from SF-6D [12]. One of the main challenges for the successful implementation of SF-6D could be related to missing data, caused by respondents not providing answers to certain items in SF-36, or being lost to follow-ups [10, 16]. Therefore, It is important to understand missingness patterns and handle missing data properly when analyzing HRQoL data, since missing data may lead to biased conclusions [17].

Normative data, data that characterize what is usual in a defined population at a specific point or period of time, are of enormous importance to primary care physicians [10, 18]. Several sets of normative values for SF-6D have been published for the general population [31–34]. Normative values for SF-6D among patients undergoing bariatric surgery can be utilized in economic evaluation and facilitate reimbursement decisions associated with treatments for patients with obesity.

The aim of this study was to establish normative values for the SF-6D index among patients undergoing bariatric surgery.

## Method

### Data

The *Scandinavian Obesity Surgery Registry (SOReg)* is a nationwide quality register of patients undergoing bariatric surgery in Sweden with a coverage exceeding 97%. It is validated regularly and is shown to have high data quality [19]. SOReg contains information on patients’ sociodemographic status, provider characteristics, details regarding procedures, and health-related outcomes, including HRQoL assessed by the SF-36 and the Obesity Problems scale (OP). HRQoL data are reported by patients at baseline and at 1, 2, and 5 years post-operatively by filling in a questionnaire. Nurses took anthropometric data and collected the completed questionnaires. Trained personnel will perform data input. For the current study, all subjects who received bariatric surgery from 2011–01-01 to 2019–03 were included (*n* = 36 706). The Ethics Authority in Sweden granted ethical permission for analyses of this study (reference number: 2019–03666).

### Health Outcome Measure

#### Short Form-36 (SF-36/RAND) and SF-6D

The SF-36 is applied in the SOReg. It measures HRQoL in eight domains, including social functioning, physical function, role-physical, bodily pain, vitality, social functioning, role-emotional, and mental health [20, 21]. The *short form six-dimensions (SF-6D)* was developed to derive a preference-based score from the SF-36 [14] or its 12-item version (SF-12) [22]. Briefly, 11 items from the original SF-36 were selected to form the six SF-6D domains (Supplementary material S2), including pain, mental health, physical functioning, social functioning, role limitations, and vitality, and each is described into four to six functional levels (Supplementary material S1). Each domain level is associated with a specific health weight. For problems reported on any level of any health domain, the corresponding weight will be deducted from the full health which is equivalent to 1 [14]. The details regarding how to calculate SF-6D can be requested from the ScHARR Outcomes team at the University of Sheffield [23]. The weights for calculating the SF-6D index are derived from a general population sample using a *standard gamble* method. The current study used the UK tariff [14] since there was no local tariff available in Sweden. Details regarding the SF-6D domains and relevant SF-36 items are given in the supplemental material (S1 and S2). The SF-6D index ranged between 0 and 1, with a higher value indicating better health.

#### Imputation of SF-6D Index

The overall missing data proportions (including both missing responses to items and missing forms due to loss to follow-up) of SF-6D in the 1-, 3-, and 5-year follow-ups were 40.7%, 62.9%, and 83.8%, respectively. The comprehensive information regarding missingness and missing patterns of the variables has been previously published [24]. Taking into consideration the longitudinal nature of the data collected, we adopted a *sequential multiple imputation (SMI)* method to impute the SF-6D index as well as other variables with missing values [24]. Briefly, the missing values of the selected eleven SF-36 items at baseline were first multiply imputed by chained equations using the baseline variables, including age, sex, BMI, pregnancy, and comorbidities. Second, for the SF-6D index of a given follow-up timepoint, missing values were imputed using the information from the baseline and previous years, including the demographic variables, SF-6D items, and comorbidities. In the current study, five imputed datasets by SMI were used to conduct descriptive and inferential analyses for the SF-6D index. The SMI method adopted in the current study might achieve an ideal imputation for the missing data even when the follow-up survey had a missing rate of 60% under the missing at random assumption [24], which could avoid significant information waste in multivariate analysis using patients with complete data only.

### Sociodemographic and Clinical Characteristics

Age was categorized into age groups of 35–44 years, 45–54 years, 55–64 years, and ≥ 65 years. Educational level was self-reported and grouped into primary school with up to 9 years of education, secondary school with 9–12 years of education, and university with over 12 years. Smoking was self-reported and categorized as yes, no, occasionally, previous smoker quit before operation, and unknown. BMI was categorized into three groups including < 40, 40–44, 45–49, and 50 kg/m^2^ and above. Comorbidities were self-reported by the patients at baseline, including the occurrence of sleep apnea, hypertension, diabetes mellitus, dyspepsia, diarrhea, depression, and other illnesses.

### Statistical Analysis

Descriptive analyses were used to examine the sample characteristics and the responses to the SF-6D domains (proportions for discrete variables, mean and standard deviation, plus median and inter-quart range for continuous variables). We applied multivariate analysis to investigate how the SF-6D index is associated with timepoint, controlling for age, sex, BMI, and comorbidities in a stepwise manner. In addition to the commonly used ordinary least squares (OLS) method, the mixed-effects Tobit regression model was used since the SF-6D index was centered at 1) [25], with random effects for patients to account for individual response clustering. The final results were combined from the five imputed datasets according to Rubin’s rule [26]. All analyses were conducted using R.4.0.2 [27] or Stata 17.0 [28].

## Results

More than 76% of the patients in the study were females, and the mean age was 41 years old at baseline. Approximately 10% of the patients were current smokers (Table 1). The mean BMI was 42 kg/m^2^ at baseline and decreased to 29 kg/m^2^ at follow-ups. In general, the presence of obesity-related comorbidities including sleep apnoea, hypertension, diabetes, dyslipidemia, and depression decreased over time and was lowest at the 1-year follow-up. HRQoL improved after surgery, with the highest improvements observed at the 1-year follow-up, followed by the 2- and 5-year follow-ups (Table 2). The SF-6D index was close to a normal distribution at baseline but left-skewed at follow-ups (Supplementary material Figure S1).

**Table 1:** Socio-demographic characteristics, at baseline and 1-, 2-, and 5-year follow-ups

	Baseline (*n* = 36,706)		1 year (*n* = 27,125)		2 years (*n* = 17,389)		5 years (*n* = 7437)	
	*n*	*%*	*n*	*%*	*n*	*%*	*n*	*%*
Age								
18–35 yrs	11,994	*32.7*	7448	*27.5*	3966	*22.8*	1179	*15.9*
36–45 yrs	11,168	*30.4*	8066	*29.7*	4876	*28.0*	1810	*24.3*
46–55 yrs	9655	*26.3*	7877	*29.0*	5405	*31.1*	2426	*32.6*
56–65 yrs	3662	*10.0*	3468	*12.8*	2817	*16.2*	1664	*22.4*
65 +	227	*0.6*	266	*1.0*	325	*1.9*	358	*4.8*
*Mean(SD)*	*41.02(11.2)*		*42.73(11.18)*		*44.59(11.22)*		*47.92(11.17)*	
*Median(Q1, Q2)*	*41(32, 49)*		*43(34, 51)*		*45(37, 53)*		*48(40, 56)*	
Sex								
Men	8637	*23.5*	6439	*23.7*	4032	*23.2*	1662	*22.3*
Women	28,069	*76.5*	20,686	*76.3*	13,357	*76.8*	5775	*77.7*
Educational level								
Missing	22,450	*61.2*	15,909	*58.7*	8920	*51.3*	3802	*51.1*
Primary school	7627	*20.8*	5999	*22.1*	4565	*26.3*	1954	*26.3*
Secondary school	1588	*4.3*	1203	*4.4*	944	*5.4*	414	*5.6*
University	5041	*13.7*	4014	*14.8*	2960	*17.0*	1267	*17.0*
Smoking								
Yes	3746	*10.2*	2669	*9.8*	1592	*9.2*	755	*10.2*
No	22,670	*61.8*	16,812	*62.0*	10,293	*59.2*	4469	*60.1*
Don’t know	3992	*10.9*	2824	*10.4*	2270	*13.1*	910	*12.2*
Occasionally	5626	*15.3*	4414	*16.3*	3013	*17.3*	1272	*17.1*
Previous smoker	670	*1.8*	406	*1.5*	221	*1.3*	31	*0.4*
BMI								
*Mean(SD)*	41.57 (5.63)		28.55 (4.56)		28.41 (4.65)		29.86 (4.93)	
*Median(Q1, Q2)*	40.8 (37.7, 44.6)		27.9 (25.3, 31.1)		27.8 (25.1, 30.9)		29.3 (26.3, 32.6)	
Comorbidity								
No	16,232	*44.2*	16,248	*59.9*	9916	*57.0*	3779	*50.8*
Yes	20,472	*55.8*	10,671	*39.3*	7140	*41.1*	3436	*46.2*
Missing	2	*0.0*	206	*0.8*	333	*1.9*	222	*3.0*
Sleep apnea								
No	32,913	*89.7*	26,046	*96.0*	16,556	*95.2*	7039	*94.6*
Yes	3791	*10.3*	873	*3.2*	500	*2.9*	176	*2.4*
Missing	2	*0.0*	206	*0.8*	333	*1.9*	222	*3.0*
Hypertension								
No	27,554	*75.1*	22,321	*82.3*	13,911	*80.0*	5643	*75.9*
Yes	9150	*24.9*	4598	*17.0*	3145	*18.1*	1572	*21.1*
Diabetes								
Missing	2	*0.0*	206	*0.8*	333	*1.9*	222	*3.0*
No	32,003	*87.2*	25,709	*94.8*	16,154	*92.9*	6744	*90.7*
Yes	4701	*12.8*	1210	*4.5*	902	*5.2*	471	*6.3*
Dyslipidemia								
Missing	2	*0.0*	206	*0.8*	333	*1.9*	222	*3.0*
No	33,213	*90.5*	25,440	*93.8*	16,018	*92.1*	6719	*90.3*
Yes	3491	*9.5*	1479	*5.5*	1038	*6.0*	496	*6.7*
Dyspepsia								
Missing	2	*0.0*	206	*0.8*	333	*1.9*	222	*3.0*
No	32,756	*89.2*	24,914	*91.8*	15,596	*89.7*	6545	*88.0*
Yes	3948	*10.8*	2005	*7.4*	1460	*8.4*	670	*9.0*
Diarrhea								
No	36,117	*98.4*	26,581	*98.0*	16,760	*96.4*	7008	*94.2*
Yes	587	*1.6*	338	*1.2*	296	*1.7*	207	*2.8*
Missing	2	*0.0*	206	*0.8*	333	*1.9*	222	*3.0*
Depression								
No	30,707	*83.7*	23,257	*85.7*	14,521	*83.5*	5959	*80.1*
Yes	5997	*16.3*	3662	*13.5*	2535	*14.6*	1256	*16.9*
Missing	2	*0.0*	206	*0.8*	333	*1.9*	222	*3.0*
Other illnesses that contributed to the surgical decision								
No	32,723	*89.1*	25,588	*94.3*	16,407	*94.4*	6948	*93.4*
Yes	3983	*10.9*	1354	*5.0*	700	*4.0*	307	*4.1*
Missing	0	*0.0*	183	*0.7*	282	*1.6*	182	*2.4*
Year								
2011	5625	*15.3*	4721	*17.4*	3582	*20.6*	2991	*40.2*
2012	5426	*14.8*	4439	*16.4*	3047	*17.5*	2189	*29.4*
2013	5399	*14.7*	4373	*16.1*	3199	*18.4*	1628	*21.9*
2014	4886	*13.3*	3865	*14.2*	2738	*15.7*	578	*7.8*
2015	4462	*12.2*	3734	*13.8*	2165	*12.5*	19	*0.3*
2016	4142	*11.3*	2697	*9.9*	1871	*10.8*	17	*0.2*
2017	3225	*8.8*	2245	*8.3*	778	*4.5*	7	*0.1*
2018	2522	*6.9*	1046	*3.9*	6	*0.0*	7	*0.1*
2019	1019	*2.8*	5	*0.0*	3	*0.0*	1	*0.0*

**Table 2:** Mean (SD) of SF-6D index by age group, with/without complication, for men and women, respectively

	SF-6D index based on imputed data (mean + SD)											
	Total				Without complication				With complication			
	Baseline	1 year	2 years	5 years	Baseline	1 year	2 years	5 years	Baseline	1 year	2 years	5 years
Women												
Age group												
18–34	0.67 ± 0.13	0.82 ± 0.13	0.79 ± 0.14	0.66 ± 0.19	0.67 ± 0.13	0.82 ± 0.13	0.79 ± 0.14	0.66 ± 0.19	0.67 ± 0.12	0.81 ± 0.14	0.78 ± 0.14	0.63 ± 0.18
35–44	0.69 ± 0.13	0.81 ± 0.14	0.79 ± 0.14	0.67 ± 0.19	0.69 ± 0.13	0.81 ± 0.14	0.79 ± 0.14	0.67 ± 0.19	0.68 ± 0.12	0.81 ± 0.14	0.80 ± 0.14	0.68 ± 0.18
45–54	0.68 ± 0.14	0.81 ± 0.14	0.79 ± 0.14	0.66 ± 0.18	0.68 ± 0.14	0.81 ± 0.14	0.80 ± 0.14	0.66 ± 0.19	0.67 ± 0.13	0.77 ± 0.16	0.77 ± 0.15	0.66 ± 0.18
55–64	0.69 ± 0.13	0.80 ± 0.14	0.79 ± 0.14	0.66 ± 0.19	0.69 ± 0.13	0.80 ± 0.14	0.79 ± 0.14	0.66 ± 0.19	0.68 ± 0.14	0.79 ± 0.15	0.77 ± 0.15	0.66 ± 0.19
65 +	0.69 ± 0.14	0.79 ± 0.14	0.78 ± 0.15	0.65 ± 0.18	0.69 ± 0.14	0.79 ± 0.14	0.78 ± 0.15	0.65 ± 0.18	0.67 ± 0.12	0.76 ± 0.12	0.74 ± 0.16	0.69 ± 0.17
Men												
Age group												
18–34	0.65 ± 0.12	0.79 ± 0.14	0.76 ± 0.14	0.63 ± 0.18	0.65 ± 0.12	0.79 ± 0.13	0.76 ± 0.14	0.63 ± 0.18	0.63 ± 0.12	0.77 ± 0.15	0.75 ± 0.15	0.64 ± 0.18
35–44	0.66 ± 0.13	0.80 ± 0.14	0.77 ± 0.14	0.65 ± 0.18	0.66 ± 0.13	0.8 ± 0.14	0.78 ± 0.14	0.65 ± 0.18	0.65 ± 0.13	0.77 ± 0.15	0.76 ± 0.15	0.65 ± 0.18
45–54	0.65 ± 0.13	0.79 ± 0.14	0.77 ± 0.15	0.64 ± 0.18	0.66 ± 0.13	0.8 ± 0.14	0.77 ± 0.15	0.64 ± 0.18	0.64 ± 0.13	0.77 ± 0.15	0.75 ± 0.15	0.64 ± 0.18
55–64	0.66 ± 0.13	0.79 ± 0.14	0.77 ± 0.14	0.64 ± 0.18	0.66 ± 0.13	0.79 ± 0.14	0.77 ± 0.14	0.64 ± 0.18	0.65 ± 0.14	0.78 ± 0.14	0.76 ± 0.14	0.65 ± 0.17
65 +	0.66 ± 0.14	0.78 ± 0.14	0.77 ± 0.14	0.63 ± 0.19	0.66 ± 0.14	0.78 ± 0.14	0.77 ± 0.14	0.63 ± 0.19	0.65 ± 0.13	0.76 ± 0.15	0.77 ± 0.16	0.65 ± 0.18

**Fig. 1:**
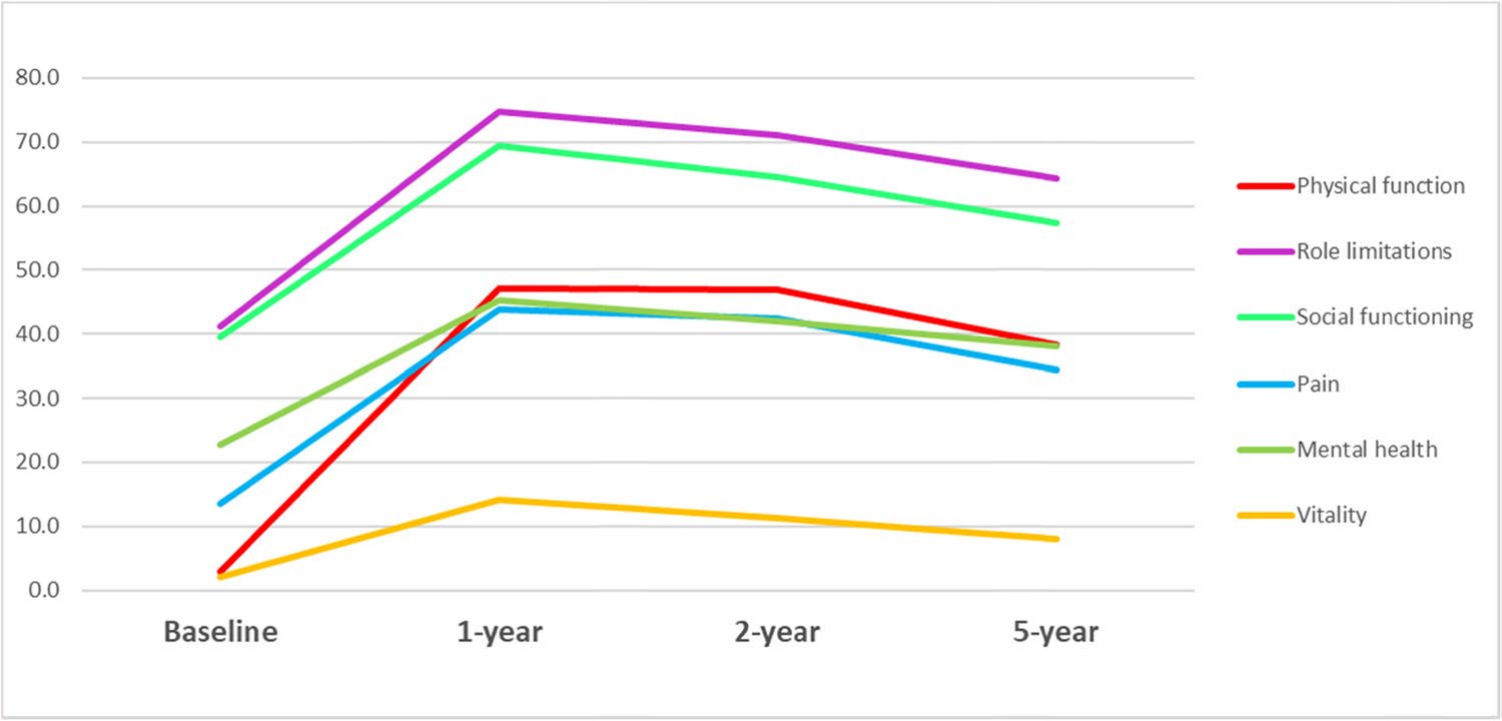
Proportion of patients without any problems on any SF-6D dimension

The proportion of patients without any problems on any SF-6D dimension is reported in Fig. 1. Improvements at the 1-year follow-up were reported in all dimensions, the largest being physical function (44%), followed by role limitations (34%), social function (30%), pain (30%), mental health (22%), and vitality (12%). At the 2- and 5-year follow-ups, a slight decrease in HRQoL relative to year 1 can be observed in all dimensions. However, relative to the baseline, there were still improvements in all dimensions. The proportions of patients reporting problems at all levels on each SF-6D dimension are presented in Figure S2 in Supplementary material S4.

The normative values for the mean and median SF-6D index, based on imputed data, are reported in Table 2 and (Supplementary material S3), respectively. The results were reported by timepoint and surgical complications for men and women, respectively. The SF-6D index increased at 1 year relative to baseline and was roughly maintained at the same level at 2 years. No normative values are presented at 5-year follow-up due to many missing data. In general, patients with complications had a slightly lower SF-6D index, and women had a slightly higher index value; however, such differences were not significant. No clear age gradient was observed.

The results from the regression analyses are reported in Table 3. Significant increases in the SF-6D index were observed at the 1- and 2-year follow-ups, with the largest increase at the 1-year follow-up. This effect was approximately the same, even after controlling for age, sex, and baseline BMI (models 2 to 4), and reduced after controlling for comorbidities (model 5). Being female was positively associated with the SF-6D index, and sleep apnea, dyspepsia, diarrhea, and depression were significantly negatively associated with the SF-6D index. No clear age gradient was observed.

**Table 3:** Multivariate analysis

	Random effect ordinary least squares (OLS)							
	Model 1 (*n* = 46,661)		Model 2 (*n* = 46,661)		Model 3 (*n* = 46,661)		Model 4 (*n* = 46,661)	
	Coefficient	*p-*value	Coefficient	*p-*value	Coefficient	*p-*value	Coefficient	*p-*value
Time point								
1 year	0.137 (0.135, 0.139)	** <** 0.001	0.137 (0.135, 0.139)	** < **0.001	0.137 (0.135, 0.139)	** <** 0.001	0.132 (0.130, 0.134)	** < **0.001
2 years	0.114 (0.112, 0.117)	** < **0.001	0.114 (0.112, 0.117)	** < **0.001	0.114 (0.112, 0.117)	** < **0.001	0.109 (0.106, 0.111)	** < **0.001
5 years	− 0.010 (− 0.016, − 0.003)	0.014	− 0.010 (− 0.016, − 0.003)	0.014	− 0.010 (− 0.016, − 0.003)	0.014	− 0.016 (− 0.022, − 0.010)	0.001
Age								
35–44 yrs			0.011 (0.008, 0.013)	** < **0.001	0.010 (0.008, 0.013)	** < **0.001	0.013 (0.012, 0.016)	** < **0.001
45–54 yrs			0.006 (0.003, 0.009**)**	** < **0.001	0.005 (0.003, 0.008)	** < **0.001	0.010 (0.008, 0.013)	** < **0.001
55–64 yrs			0.006 (0.001, 0.010)	0.014	0.005 (0.001, 0.009)	0.025	0.011 (0.006, 0.015)	< 0.001
65 +			− 0.002 (− 0.012, 0.008)	0.698	− 0.003 (− 0.013, 0.008)	0.585	0.004 (− 0.006, 0.015)	0.418
Sex								
Women			0.021 (0.019, 0.023)	** <** 0.001	0.022 (0.020, 0.024)	** <** 0.001	0.018 (0.015, 0.020)	** < **0.001
BMI								
40–44					0.001 (− 0.002, 0.003)	0.577	0.001 (− 0.002, 0.004)	0.473
45–49					− 0.003(− 0.007, 0.001)	0.089	− 0.003 (− 0.007, 0.001)	0.116
50 +					− 0.012 (− 0.017, − 0.007)	**<** 0.001	− 0.010 (− 0.015, − 0.005)	0.001
Comorbidity								
Sleep apnea							− 0.008 (− 0.012, − 0.004)	** < **0.001
Hypertension							0.000 (− 0.003. 0.003)	0.935
Diabetes							0.001 (− 0.002, 0.005)	0.537
Dyslipidemia							− 0.000 (− 0.005, 0.004)	0.871
Dyspepsia							− 0.020 (− 0.023, − 0.017)	**<** 0.001
Diarrhea							− 0.020 (− 0.027, − 0.013)	** < **0.001
Depression							− 0.054 (− 0.057, − 0.050)	** <** 0.001
Other							− 0.033 (− 0.038, − 0.029)	** < **0.001
Goodness of fit								
* F*	3144.3		2143.83		1726.1		1323.71	
* p*-value	< 0.001		< 0.001		< 0.001		< 0.001	
	Random effect Tobit regression							
	Model 1 (*n* = xx)		Model 2 (*n* = xx)		Model 3 (*n* = xx)		Model 4 (*n* = xx)	
	Coefficient	*p-*value	Coefficient	*p-*value	Coefficient	*p-*value	Coefficient	*p-*value
Time point								
1 year	0.137 (0.135, 0.139)	** < **0.001	0.137 (0.135, 0.139)	** < **0.001	0.137 (0.135, 0.139)	** < **0.001	0.132 (0.130, 0.134)	** <** 0.001
2 years	0.115 (0.112, 0.117)	** < **0.001	0.115 (0.112, 0.117)	** < **0.001	0.114 (0.112, 0.117)	** <** 0.001	0.109 (0.106, 0.111)	** <** 0.001
5 years	− 0.010 (− 0.017, − 0.003)	0.013	− 0.010 (− 0.017, − 0.003)	0.013	− 0.010 (− 0.017, − 0.003)	0.013	− 0.016 (− 0.023, − 0.010)	0.001
Age								
35–44 yrs			0.011 (0.008, 0.013)	** < **0.001	0.010 (0.008, 0.013)	** < **0.001	0.013 (0.012, 0.016)	** < **0.001
45–54 yrs			0.006 (0.003, 0.009)	** < **0.001	0.006 (0.003, 0.008)	** < **0.001	0.010 (0.008, 0.013)	** < **0.001
55–64 yrs			0.006 (0.001, 0.010)	0.013	0.005 (0.001, 0.009)	0.024	0.011 (0.006, 0.015)	< 0.001
65 +			− 0.002 (− 0.013, 0.009)	0.706	− 0.003 (− 0.013, 0.008)	0.595	0.004 (− 0.006, 0.015)	0.415
Sex								
Women			0.021 (0.019, 0.023)	** <** 0.001	0.022 (0.020, 0.024)	** < **0.001	0.018 (0.015, 0.020)	** <** 0.001
BMI								
40–44					0.001 (− 0.002, 0.003)	0.574	0.001 (− 0.002, 0.004)	0.471
45–49					− 0.003(− 0.007, 0.001)	0.093	− 0.003 (− 0.007, 0.001)	0.12
50 +					** − **0.012 (− 0.017, − 0.007)	** < **0.001	** − **0.010 (− 0.015, − 0.005)	0.001
Comorbidity								
Sleep apnea							** − **0.008 (− 0.012, − 0.004)	** < **0.001
Hypertension							0.000 (− 0.003. 0.003)	0.931
Diabetes							0.001 (− 0.002, 0.005)	0.556
Dyslipidemia							− 0.000 (− 0.005, 0.004)	0.868
Dyspepsia							** − **0.020 (− 0.023, − 0.017)	** < **0.001
Diarrhea							** − **0.020 (− 0.027, − 0.013)	** <** 0.001
Depression							** − **0.054 (− 0.058, − 0.051)	** < **0.001
Other							** − **0.034 (− 0.038, − 0.029)	** < **0.001
Goodness of fit								
* F*	3116.26		2128.48		1715.1		1316.12	
* p*-value	< 0.001		< 0.001		< 0.001		< 0.001	

Reference group

Baseline

18–35 yrs

Men

BMI < 039

No disease

## Discussion

To the best of our knowledge, this study has established the first normative values for SF-6D among patients undergoing bariatric surgery. The results of the study can be used in economic evaluations for bariatric surgery. The results also demonstrate significant improvements in HRQoL following bariatric surgery in all health dimensions. This finding supports previous studies reporting that HRQoL improves after bariatric surgery, especially for dimensions related to physical function [12, 13, 29].

Surprisingly, almost no study has assessed the SF-6D after bariatric surgery despite that SF-36 has been extensively applied among such patients [12, 29]. Economic evaluations of patients undergoing bariatric often applied health preferences derived from other PBM, such as EQ-5D, or mapping algorithms [30]. Our study has demonstrated that the SF-6D health preferences can be derived from the SF-36 with relative ease, and we hope this work might raise awareness that large HRQoL databases on bariatric surgery such as the one generated by SOReg, which reports SF-36 data, can be better utilized. The SF-6D has a particular strength in that it can be derived from the SF-36 specifically for use in economic evaluation, which can inform decisions on reimbursement of health technology in the treatment of people with obesity. This study has the potential to improve healthcare economic data from previous studies, by converting SF-36 into SF-6D in patients undergoing bariatric surgery. Comparing our results with the SF-6D norms for the general population [31–34], the patients undergoing bariatric surgery reported lower SF-6D index at baseline, but almost the same level as the general population at 1- and 2-year follow-ups. This suggests that bariatric surgery improves HRQoL for patients with obesity to almost the level of a normal population supporting the benefits not only in terms of cardiometabolic effects but also in terms of patients-reported outcome translating into direct values to the individual. A previous systematic review found no consistency in the literature concerning long-term HRQoL after bariatric surgery. Some research indicates that HRQoL improves for up to 1 year and then plateaus and/or declines [31, 32], while other research suggested that HRQoL continues to improve after surgery [33]. In our study, we found that the greatest improvement in HRQoL was at 1 year, which declined slightly at 2 years but still significantly improved relative to the baseline. Unfortunately, we cannot draw any conclusions about HRQoL at 5 years after surgery due to the many patients lost at follow-up after 5 years.

### Strength and Limitations

The main strength of our study was the use of real-world data from a large national patient register, which enables us to report HRQoL changes over time before and after bariatric surgery, as well as the influence of surgery on different health domains. Normative data may be obtained in cross-sectional studies or longitudinal study. As we do not expect potential epoch effects associated with bariatric surgery and to maximize the sample size, we pulled data from different years together. We also applied multivariate analyses to examine whether the improvement remained after adjusting for age, sex, BMI, and comorbidities. To the best of our knowledge, this is the first study that reported SF-6D normative values among bariatric patients. Such data can be utilized in economic evaluation. Nevertheless, the SF-36 scores would be still useful, especially in the clinical settings. The SMI method adopted in the current study might be an ideal strategy for handling missing data (even though the follow-up survey had a missing proportion of 60%), avoiding significant information waste in the multivariate analysis.

There are a few limitations that need to be addressed: we based our calculation of SF-6D on the UK value set, as there was not a Swedish one. People in different countries may value health differently; however, as both are European countries, we do not expect that people will value health very differently. Some surgical centers had a low response rate HRQoL, but since most centers in Sweden have similar characteristics in patient cohorts, this is unlikely to have a significant impact on the representativeness of our study sample. Moreover, due to the high missingness at 5 years (80%), even the SMI could not handle this, so we are not able to draw a conclusion about HRQoL beyond 2 years from the baseline. Data quality for self-reported educational level is low, and therefore, socio-economic status could not be included in the analyses. To prevent and handle missing data in HRQoL studies, researchers should apply a rigorous methodology and practices. Guidance for preventing and handling missing data in observational studies is needed, and studies that use real-world data should be prioritized. For the analysis of socio-economic status, linkage with national registries is required. A previous study which is also based on SOReg (with linkage data for socio-economic status) investigated the association between weight loss and socio-economic status and has shown that the first generation immigrants and patients residing in larger towns (> 200,000 inhabitants) tend to have inferior weight loss compared to other groups; however, the remaining socioeconomic factors appear to have a weaker association with postoperative weight loss [34].

## Conclusions

This study established normative values for SF-6D among patients undergoing bariatric surgery based on a large registry. The results of the study can be used in economic evaluations for bariatric surgery.

### Supplementary Information

Below is the link to the electronic supplementary material.

Supplementary file1 (PDF 591 KB)

## Data Availability

Data sharing is not possible according to Swedish law.
