# Supplementary material for: SF-6D Normative Values Among Patients Undergoing Bariatric Surgery: Results Based on Real-World Evidence from the Scandinavian Obesity Surgery Registry (SOReg)
Source: Obes Surg. 2024 Jan 8;34(2):558–67. doi: 10.1007/s11695-023-07024-0 (PMC10811135; doi:10.1007/s11695-023-07024-0)

SF-6D Normative values among Patients with Bariatric patients: Results based on real-world evidence from the Scandinavian Obesity Surgery Registry (SOReg)

Supplementary Material

S1. The short form-6D (SF-6D)[a]

**Physical functioning**		
	1	Your health does not limit you in vigorous activities
	2	Your health limits you a little in vigorous activities
	3	Your health limits you a little in moderate activities
	4	Your health limits you a lot in moderate activities
	5	Your health limits you a little in bathing and dressing
	6	Your health limits you a lot in bathing and dressing
**Role limitations**		
	1	You have no problems with your work or other regular daily activities as a result of your physical health or any emotional problems
	2	You are limited in the kind of work or other activities as a result of your physical health
	3	You accomplish less than you would like as a result of emotional problems
	4	You are limited in the kind of work or other activities as a result of your physical health and accomplish less than you would like as a result of emotional problems
**Social functioning**		
	1	Your health limits your social activities none of the time
	2	Your health limits your social activities a little of the time
	3	Your health limits your social activities some of the time
	4	Your health limits your social activities most of the time
	5	Your health limits your social activities all of the time
**Pain**		
	1	You have no pain
	2	You have pain but it does not interfere with your normal work (both outside the home and housework)
	3	You have pain that interferes with your normal work (both outside the home and housework) a little bit
	4	You have pain that interferes with your normal work (both outside the home and housework) moderately
	5	You have pain that interferes with your normal work (both outside the home and housework) quite a bit
	6	You have pain that interferes with your normal work (both outside the home and housework) extremely
**Mental health**		
	1	You feel tense or downhearted and low none of the time
	2	You feel tense or downhearted and low a little of the time
	3	You feel tense or downhearted and low some of the time
	4	You feel tense or downhearted and low most of the time
	5	You feel tense or downhearted and low all of the time
**Vitality**		
	1	You have a lot of energy all of the time

	2	You have a lot of energy most of the time
	3	You have a lot of energy some of the time
	4	You have a lot of energy a little of the time
	5	You have a lot of energy none of the time

[a] The SF-36 items used to construct the SF-6D are as follows: physical functioning items 1, 2 and 10; role limitation due to physical problems item 3; role limitation due to emotional problems item 2; social functioning item 2; both bodily pain items; mental health items 1 (alternate version) and 4; and vitality item 2.

S2. SF-36 items used to construct the SF-6D

**SF-6D domain**	**SF-36 item**	
**Physical function**	**pf1**	The following items are about activities you might do during a typical day. Does your health now limit you in these activities? If so, how much? Vigorous activities, such as running, lifting heavy objects, participating in strenuous sports.
	**pf2**	The following items are about activities you might do during a typical day. Does your health now limit you in these activities? If so, how much? Moderate activities, such as moving a table, pushing a vacuum cleaner, bowling, or playing golf
	**pf10**	The following items are about activities you might do during a typical day. Does your health now limit you in these activities? If so, how much? Bathing or dressing yourself
**Role participation**	**rp3**	During the past 4 weeks, have you had any of the following problems with your work or other regular daily activities as a result of your physical health?<br>Were limited in the kind of work or other activities
	**re2**	During the past 4 weeks, have you had any of the following problems with your work or other regular daily activities as a result of any emotional problems (such as feeling depressed or anxious)? Accomplished less than you would like
**Social function**	**sf2**	During the past 4 weeks, how much of the time has your physical health or emotional problems interfered with your social activities (like visiting with friends, relatives, etc.)
**Bodily pain**	**bp1**	How much bodily pain have you had during the past 4 weeks?
	**bp2**	During the past 4 weeks, how much did pain interfere with your normal work (including both work outside the home and housework)?
**Mental health**	**mh1**	These questions are about how you feel and how things have been with you during the past 4 weeks. For each question, please give the one answer that comes closest to the way you have been feeling. How much of the time during the past 4 weeks…Have you been a very nervous person?
	**mh4**	These questions are about how you feel and how things have been with you during the past 4 weeks. For each question, please give the one answer that comes closest to the way you have been feeling. How much of the time during the past 4 weeks…Have you felt downhearted and blue?
**Vitality**	**vt2**	These questions are about how you feel and how things have been with you during the past 4 weeks. For each question, please give the one answer that comes closest to the way you have been feeling. How much of the time during the past 4 weeks…Did you have a lot of energy?

S3. Median (SD) of SF-6D index by age group, with/without complication, for men and women, respectively.

	SF-6D index											
	Total				Without complication				With complication			
	Baseline	1-year	2-year	5-year	Baseline	1-year	2-year	5-year	Baseline	1-year	2-year	5-year
Age group	Women											
18-34	0.67,[0.58, 0.77]	0.85,[0.73, 0.92]	0.83,[0.67, 0.89]	0.63,[0.5, 0.84]	0.67,[0.58, 0.77]	0.85,[0.73, 0.92]	0.83,[0.67, 0.89]	0.64,[0.51, 0.84]	0.66,[0.59, 0.75]	0.85,[0.71, 0.89]	0.83,[0.67, 0.89]	0.61,[0.49, 0.76]
35-44	0.68,[0.59, 0.8]	0.85,[0.72, 0.92]	0.84,[0.67, 0.89]	0.65,[0.51, 0.84]	0.68,[0.59, 0.8]	0.85,[0.72, 0.92]	0.84,[0.67, 0.89]	0.66,[0.51, 0.84]	0.67,[0.59, 0.79]	0.85,[0.72, 0.93]	0.85,[0.69, 0.89]	0.65,[0.54, 0.85]
45-54	0.67,[0.59, 0.8]	0.85,[0.71, 0.92]	0.84,[0.67, 0.89]	0.64,[0.51, 0.83]	0.68,[0.59, 0.8]	0.85,[0.72, 0.93]	0.84,[0.68, 0.89]	0.64,[0.51, 0.84]	0.65,[0.58, 0.77]	0.81,[0.66, 0.89]	0.81,[0.64, 0.89]	0.64,[0.54, 0.83]
55-64	0.67,[0.59, 0.8]	0.85,[0.7, 0.89]	0.83,[0.67, 0.89]	0.65,[0.51, 0.84]	0.67,[0.59, 0.8]	0.85,[0.7, 0.89]	0.83,[0.67, 0.89]	0.65,[0.51, 0.84]	0.69,[0.59, 0.79]	0.84,[0.67, 0.89]	0.81,[0.64, 0.89]	0.65,[0.52, 0.84]
65+	0.67,[0.57, 0.81]	0.84,[0.68, 0.89]	0.83,[0.67, 0.89]	0.66,[0.5, 0.83]	0.68,[0.57, 0.83]	0.84,[0.68, 0.89]	0.84,[0.67, 0.89]	0.64,[0.49, 0.81]	0.66,[0.58, 0.76]	0.78,[0.68, 0.85]	0.81,[0.61, 0.85]	0.72,[0.58, 0.84]
	Men											
Age group												
18-34	0.63,[0.56, 0.73]	0.83,[0.68, 0.89]	0.8,[0.64, 0.89]	0.62,[0.49, 0.81]	0.64,[0.56, 0.73]	0.83,[0.68, 0.89]	0.8,[0.64, 0.89]	0.62,[0.49, 0.81]	0.62,[0.55, 0.72]	0.82,[0.65, 0.89]	0.77,[0.62, 0.89]	0.62,[0.49, 0.81]
35-44	0.64,[0.56, 0.75]	0.84,[0.69, 0.89]	0.81,[0.65, 0.89]	0.64,[0.5, 0.83]	0.64,[0.57, 0.75]	0.84,[0.7, 0.89]	0.81,[0.65, 0.89]	0.64,[0.5, 0.83]	0.62,[0.56, 0.75]	0.81,[0.65, 0.89]	0.8,[0.64, 0.89]	0.64,[0.5, 0.83]
45-54	0.64,[0.56, 0.75]	0.84,[0.68, 0.89]	0.81,[0.64, 0.89]	0.62,[0.49, 0.81]	0.64,[0.56, 0.75]	0.84,[0.69, 0.89]	0.81,[0.65, 0.89]	0.62,[0.49, 0.81]	0.62,[0.56, 0.74]	0.81,[0.65, 0.89]	0.77,[0.62, 0.89]	0.62,[0.5, 0.81]
55-64	0.64,[0.56, 0.77]	0.83,[0.68, 0.89]	0.81,[0.65, 0.89]	0.62,[0.5, 0.81]	0.64,[0.56, 0.77]	0.83,[0.69, 0.89]	0.81,[0.65, 0.89]	0.62,[0.5, 0.81]	0.63,[0.55, 0.76]	0.81,[0.66, 0.89]	0.78,[0.64, 0.85]	0.62,[0.5, 0.81]
65+	0.64,[0.56, 0.78]	0.81,[0.69, 0.89]	0.81,[0.65, 0.89]	0.6,[0.48, 0.81]	0.64,[0.56, 0.79]	0.82,[0.69, 0.89]	0.82,[0.66, 0.89]	0.61,[0.47, 0.81]	0.62,[0.56, 0.75]	0.8,[0.62, 0.89]	0.79,[0.65, 0.86]	0.59,[0.52, 0.81]

S4. Proportions of patients reporting problems at SF-6D dimensions

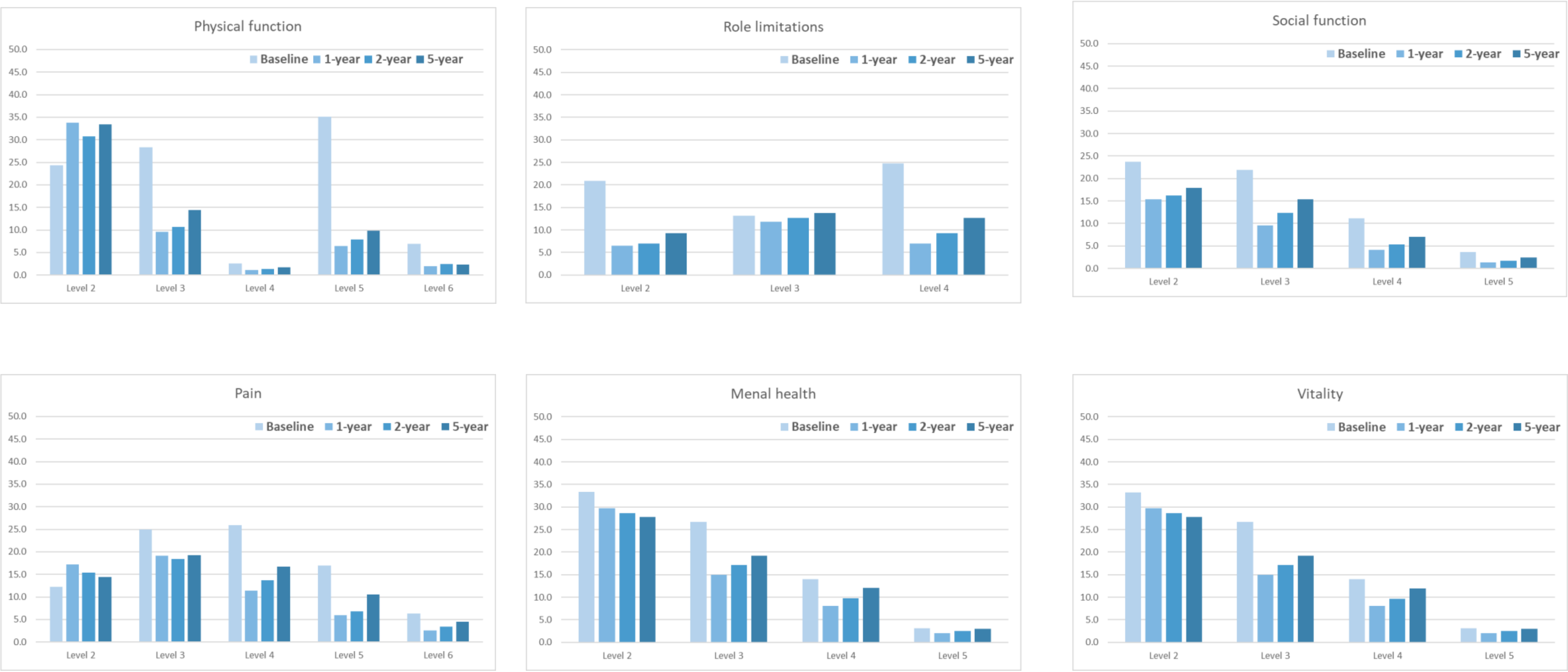

Supplement: Supplementary file 1 — Supplementary file1 (PDF 591 KB) [file 11695_2023_7024_MOESM1_ESM.pdf]
